# Transcranial Doppler ultrasound in the ICU: it is not all sunshine and rainbows

**DOI:** 10.1186/s13089-018-0085-4

**Published:** 2018-01-16

**Authors:** Pablo Blanco, Anselmo Abdo-Cuza

**Affiliations:** 1 Ecodiagnóstico-Centro de Diagnóstico por Imágenes, 3272, 50 St., Necochea, 7630 Argentina; 2 Centro de Investigaciones Médico-Quirúrgicas, 11-13 and 216 St., Siboney, La Habana, 12100 Cuba

Dear Editor,

We read the article about Transcranial Doppler (TCD) for intensivists [1]. Although not a novel ultrasound technique, in particular the “blind” o non-imaging TCD (bTCD), authors´ efforts to promote some basic applications of the Duplex technique (transcranial color-coded duplex sonography, TCCS) are remarkable.

However, some technical points and assertions are dubious and/or incorrect, as noted below:

In the first place, regarding the midline shift (MLS) measurement technique by TCCS, (A-B)/2 is well-studied and validated against CT [2]. While proposed by authors’ as an “internal standard” [1], as shown in Fig. 1 of the original article [1], measuring the distance to the contralateral cranial bone is not described in the original technique, it is unnecessary and adds complexity; thus, it should not be taken into account, as is the case with the mentioned “C and D” technique. To the authors´ knowledge, whether methodologically correct or not, there are no study validating either of them. Practitioners should be aware that the MLS measurement by TCCS is not reliable in the presence of bone defects (like decompressive craniectomy or skull fractures), temporal cephalohematoma, or changes in intracranial anatomy secondary to trauma [3], citing the most common examples observed in daily practice. Particularly in patients with a decompressive craniectomy, an alternative MLS measurement technique is well validated against CT [4].

**Fig. 1:**
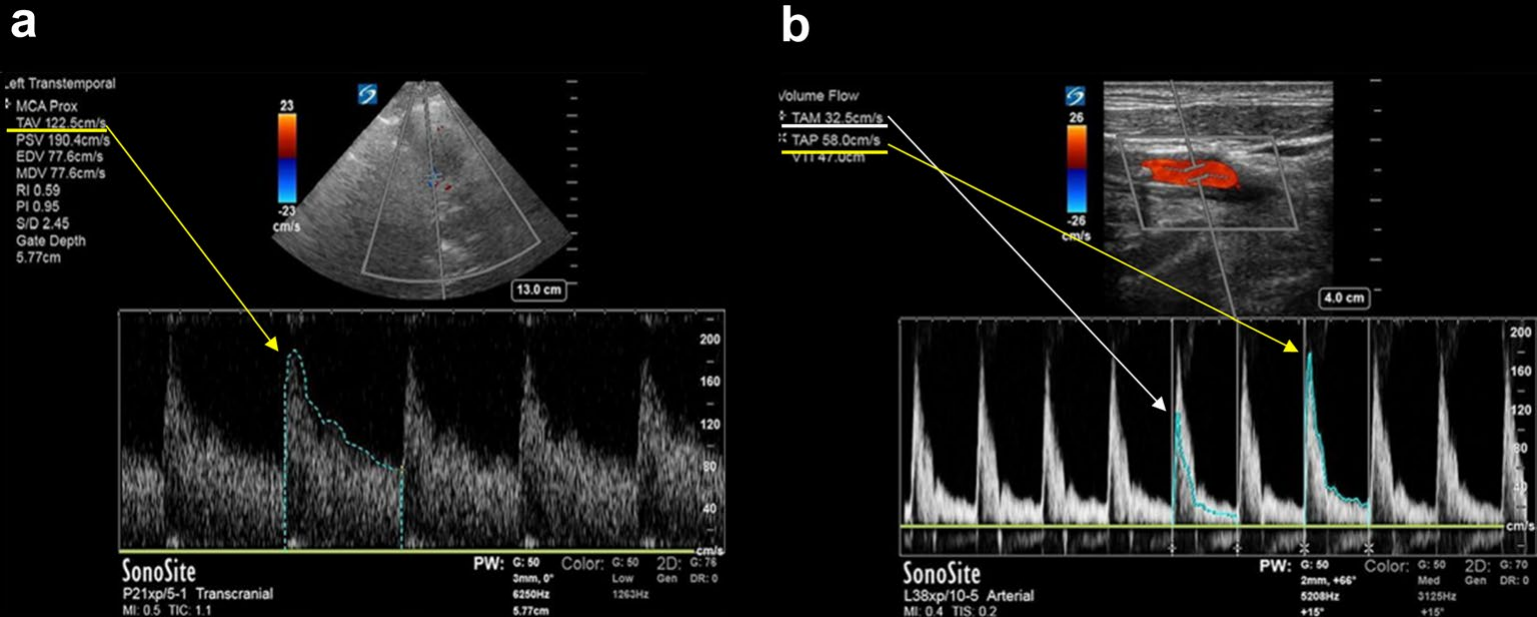
(corresponding to b and c of Fig. 2 [1]). Note the different and confusing nomenclature regarding “mean velocities”. As depicted from the trace of the envelope of the Doppler spectra (yellow arrows), time-averaged maximum velocity is recorded, namely, TAV (time-averaged velocity) in (**a**) and TAP (time-averaged peak velocity) in (**b**). There is also no doubt in **a** that is TAP, because pulsatility index (PI) is calculated using this value [peak systolic velocity (PSV)-end-diastolic velocity (EDV)/TAV]. Time-averaged mean velocity is not recorded in (**a**), but is shown in (**b**) as TAM, traced in the middle of the Doppler spectra (white arrow). In TCCS, time-averaged maximum or peak velocities are the “mean” velocities that should be considered. The correct Lindegaard Index (Middle cerebral artery TAP/internal carotid artery TAP) in this case is 123/58, equal to 2.1 (corresponding to hyperemia if considered independently). It is thus clearly incorrect to use different “mean velocities” when calculating the LI, such as TAP/TAMEAN. Note: the waveform in (**b**) is consistent with an external carotid artery flow, given its sharp systolic upstroke, high-resistance velocity profile, and early diastolic notch (another mistake that should be taken into account)

Second, when moving from a bTCD technique to the Duplex technique, practitioners must be aware of the “mean velocities” recorded by the ultrasound machine: time-averaged maximum velocity, known as TAMAX or TAP and time-averaged mean velocity, also known as TAMEAN or TAMV. While both are “mean” velocities, TAMEAN is approximately half the TAMAX [5]. Since in TCCS, the velocity considered is the TAMAX [5], using TAMEAN instead of TAP leads to underestimating velocities. This is clearly evidenced in Fig. 2 [1], where in the TCCS image, TAP is correctly used, but in the transcervical insonation, TAMV is used instead of TAP. Indeed, TAP should be compared when the Lindegaard Index (LI) is used, but comparing TAMAX/TAMEAN as is performed in Fig. 2 is an obvious mistake and readers need to be cautioned from making the same error. The actual LI in this case is 2.1, which indicates hyperemia (Fig. 1). According to this now well-performed TCD ratio, the angiographic finding of vasospasm was fortuitous, at least if this index is used independently [6]. In addition, transcervical insonation should be performed with the same phased-array probe to observe the “distal” extracranial internal carotid artery (ICA)—TAP (Fig. 2a) [7]. It should be noted that this segment is not assessed with the linear probe as shown in Fig. 2c of the original article. In addition, large correction angles (60°) result when a linear transducer is used and this must be especially avoided when comparing middle cerebral artery (MCA)/ICA TAP. Thus, the Doppler correction angle should not be used [8]. As noted, transcervical insonation should be a basic part of point-of-care ultrasound (POCUS)-TCD, at least if vasospasm evaluation is considered.

**Fig. 2:**
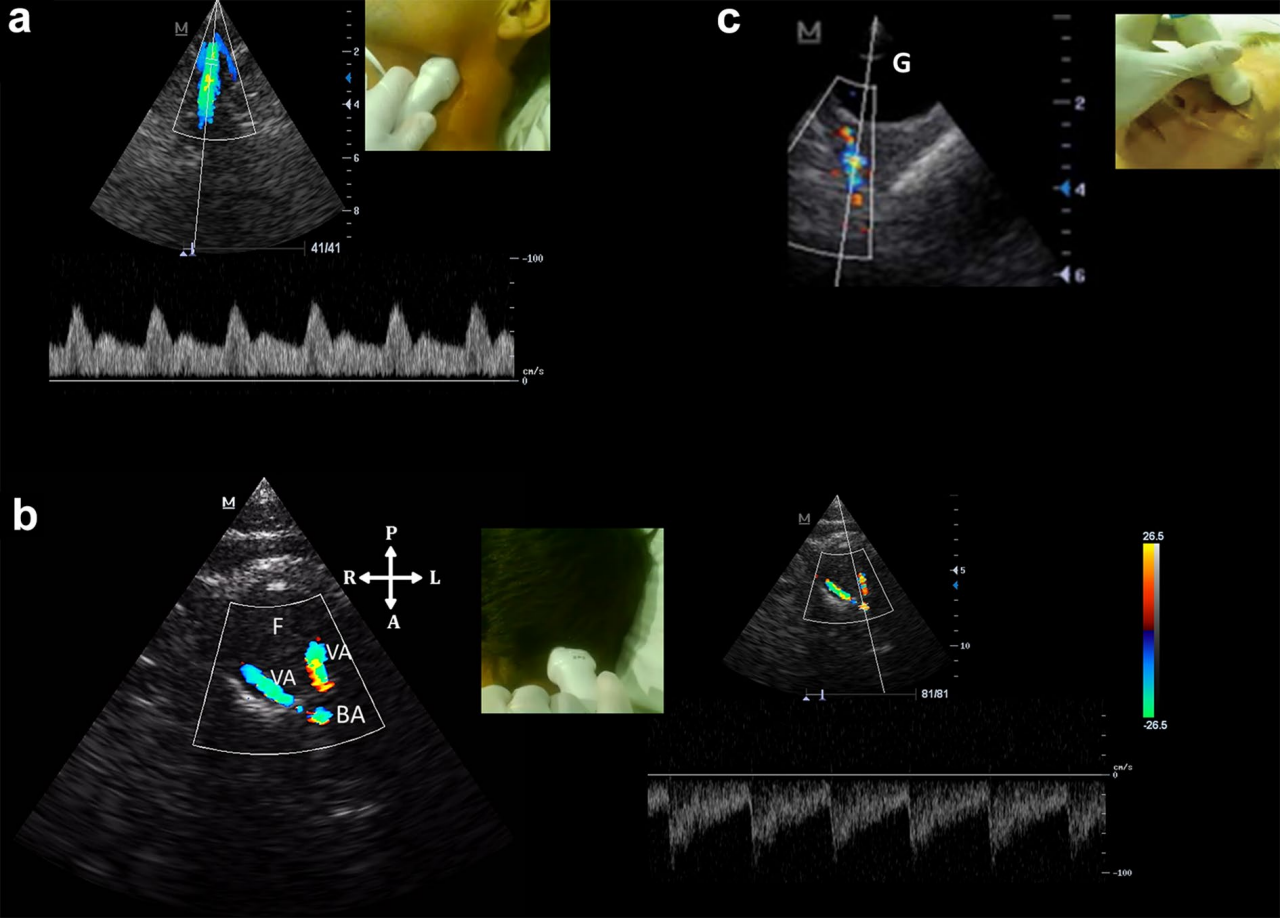
**a** Transcervical window, phased-array probe. Note that the distal internal carotid artery is insonated and that angle correction is not needed in pulsed-wave Doppler. **b** Transforaminal window, phased- array probe. Note the inverted V configuration of the posterior circulation on color Doppler imaging (coded blue, indicating that blood is moving away from the transducer), depicted by both vertebral arteries (VA) and the basilar artery (BA), showing also the corresponding spectral Doppler on the inferior channel. F: foramen magnum; *VA* vertebral artery; *BA* basilar artery. **c** Transorbital window, phased-array probe. *G*: ocular globe

Third, to the best of our knowledge, we are not aware of any guidelines that recommend TCD as a screening tool for further indication of an ancillary test to confirm the diagnosis of brain death. When determining the presence of cerebral circulatory arrest (CCA), many countries around the world accept this tool as an ancillary test to confirm the clinical diagnosis of brain death [9]. For example, there are formal TCD guidelines in Latin-American addressing this issue [10, 11]. For this indication, accepted TCD-CCA criteria for both “anterior” and “posterior” cerebral arterial circulation must be registered [12, 13]. Thus, intuitively, the transtemporal window is not enough for this indication. As a point-of-care application, transforaminal window should also be considered a basic window, at least if a CCA application is proposed (Fig. 2b). Transorbital (Fig. 2c) and transcervical (Fig. 2a) are also useful (although not fully accepted) in some actual patients to determine CCA, in particular when intracranial arterial flows are not detected on first examination, due to inadequate bone insonation windows, for example (observed in at least 25% of the patients) [13]. Regarding Doppler CCA criteria, the oscillating flow, although a biphasic flow, needs to be clearly differentiated from a high-resistance biphasic flow with a net forward flow (Fig. 3). In doubtful cases, always correlating with the clinical signs of brain death, modifications of the waveforms with interventions, such as osmotic therapy, may allow practitioners to discard the CCA diagnosis given the reversibility of the case on follow-up examinations.

**Fig. 3:**
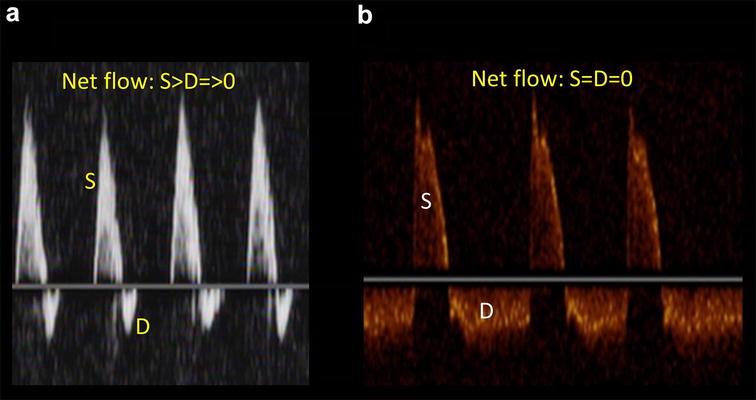
**a** High-resistance biphasic flow, with a net forward flow, not compatible with cerebral circulatory arrest. **b** Oscillating flow, with a net flow of 0, corresponding to cerebral circulatory arrest. *S* systole; *D* diastole

Finally, velocities and indices (e.g., pulsatility index) are highly variable, resulting from physiologic (arousal, for example) to pathologic conditions (e.g., raising intracranial pressure) (Tables 1 and 2). Thus, caution should be exercised when interpreting TCD findings, which should always be considered within a multimodality monitoring, and not in isolation. The phrase “trends are your friend” is highly applicable when interpreting TCD velocities and indices.

**Table 1:** Physiologic and pathologic conditions that can modify TCCS flow velocities [3]

Increase
Hyperemia
Fever, anemia, high cardiac output, arterial hypertension
Vasospasm
Intracranial arterial stenosis (for example atherosclerotic plaque)
Hypercapnia
Bacterial meningitis
Pre-eclampsia
Decrease
Raised intracranial pressure
Decreased cerebral perfusion pressure
Cerebral circulatory arrest
Hypocapnia
Hypothermia
Wrong insonation angle

**Table 2:** Physiologic and pathologic conditions that can modify TCCS Doppler indices [3]

Increase
Raised intracranial pressure
Decreased cerebral perfusion pressure
Hypocapnia
Hypothermia
Cerebral circulatory arrest
Hyperviscosity
Intracranial artery occlusion
Advanced age (vessel stiffness)
Decrease
Hyperemia
Anemia, fever, high cardiac output, arterial hypertension
Hypercapnia
Vasospasm
Intracranial artery stenosis
Arteriovenous malformation
Rewarming following hypothermia

In conclusion, POCUS TCD is not a perfect technique. Many aspects (technical and interpretative) should be considered to obtain a reliable TCD exam. In addition, for the reasons explained above, TCCS should not be limited to transtemporal windows, since transforaminal, transcervical, and transorbital windows have a defined role in basic TCD applications. The entire TCCS exam is performed with the same phased-array probe, based on the simplicity of POCUS, without the need of formal TCD examinations or dedicated machines, as happens with most (if not all) POCUS applications in the ICU. It is clear that a TCD-training curricula is mandatory to fulfill intensivists’ needs.

## Abbreviations

TCD: transcranial Doppler
bTCD: blind or non-imaging TCD
TCCS: transcranial color-coded duplex sonography
MLS: midline shift
TAMAX/TAP: time-averaged maximum velocity/time-averaged peak velocity
TAMEAN/TAMV: time-averaged mean velocity
LI: Lindegaard Index
CCA: cerebral circulatory arrest
POCUS: point-of-care ultrasound
CT: computed tomography
